# Interacting networks of resistance, virulence and core machinery genes identified by genome-wide epistasis analysis

**DOI:** 10.1371/journal.pgen.1006508

**Published:** 2017-02-16

**Authors:** Marcin J. Skwark, Nicholas J. Croucher, Santeri Puranen, Claire Chewapreecha, Maiju Pesonen, Ying Ying Xu, Paul Turner, Simon R. Harris, Stephen B. Beres, James M. Musser, Julian Parkhill, Stephen D. Bentley, Erik Aurell, Jukka Corander

**Affiliations:** 1 Department of Chemistry, Vanderbilt University, Nashville, TN, United States of America; 2 Department of Infectious Disease Epidemiology, Imperial College London, London, United Kingdom; 3 Department of Computer Science, Aalto University, Espoo, Finland; 4 Department of Medicine, University of Cambridge, Cambridge, United Kingdom; 5 Shoklo Malaria Research Unit, Mahidol-Oxford Tropical Medicine Research Unit, Faculty of Tropical Medicine, Mahidol University, Mae Sot, Thailand; 6 Centre for Tropical Medicine, Nuffield Department of Medicine, University of Oxford, Oxford, United Kingdom; 7 Pathogen Genomics, Wellcome Trust Sanger Institute, Cambridge, United Kingdom; 8 Center for Molecular and Translational Human Infectious Diseases Research, Department of Pathology and Genomic Medicine, Houston Methodist Research Institute, and Houston Methodist Hospital, Houston, Texas, United States of America; 9 Departments of Pathology and Laboratory Medicine and Microbiology and Immunology, Weill Cornell Medical College, New York, New York, United States of America; 10 Department of Computational Biology, KTH–Royal Institute of Technology, Stockholm, Sweden; 11 Departments of Applied Physics and Computer Science, Aalto University, Espoo, Finland; 12 Institute of Theoretical Physics, Chinese Academy of Sciences, Beijing, China; 13 Department of Mathematics and Statistics, University of Helsinki, Helsinki, Finland; 14 Department of Biostatistics, University of Oslo, Oslo, Norway; 15 Department of Veterinary Medicine, University of Cambridge, Cambridge, United Kingdom; National Institute of Genetics, JAPAN

## Abstract

Recent advances in the scale and diversity of population genomic datasets for bacteria now provide the potential for genome-wide patterns of co-evolution to be studied at the resolution of individual bases. Here we describe a new statistical method, genomeDCA, which uses recent advances in computational structural biology to identify the polymorphic loci under the strongest co-evolutionary pressures. We apply genomeDCA to two large population data sets representing the major human pathogens *Streptococcus pneumoniae* (pneumococcus) and *Streptococcus pyogenes* (group A Streptococcus). For pneumococcus we identified 5,199 putative epistatic interactions between 1,936 sites. Over three-quarters of the links were between sites within the *pbp2x*, *pbp1a* and *pbp2b* genes, the sequences of which are critical in determining non-susceptibility to beta-lactam antibiotics. A network-based analysis found these genes were also coupled to that encoding dihydrofolate reductase, changes to which underlie trimethoprim resistance. Distinct from these antibiotic resistance genes, a large network component of 384 protein coding sequences encompassed many genes critical in basic cellular functions, while another distinct component included genes associated with virulence. The group A Streptococcus (GAS) data set population represents a clonal population with relatively little genetic variation and a high level of linkage disequilibrium across the genome. Despite this, we were able to pinpoint two RNA pseudouridine synthases, which were each strongly linked to a separate set of loci across the chromosome, representing biologically plausible targets of co-selection. The population genomic analysis method applied here identifies statistically significantly co-evolving locus pairs, potentially arising from fitness selection interdependence reflecting underlying protein-protein interactions, or genes whose product activities contribute to the same phenotype. This discovery approach greatly enhances the future potential of epistasis analysis for systems biology, and can complement genome-wide association studies as a means of formulating hypotheses for targeted experimental work.

## Author summary

Epistatic interactions between polymorphisms in DNA are recognized as important drivers of evolution in numerous organisms. Study of epistasis in bacteria has been hampered by the lack of densely sampled population genomic data, suitable statistical models and inference algorithms sufficiently powered for extremely high-dimensional parameter spaces. We introduce the first model-based method for genome-wide epistasis analysis and use two of the largest available bacterial population genome data sets on *Streptococcus pneumoniae* (the pneumococcus) and *Streptococcus pyogenes* (group A *Streptococcus*) to demonstrate its potential for biological discovery. Our approach reveals interacting networks of resistance, virulence and core machinery genes in the pneumococcus, which highlights putative candidates for novel drug targets. We also discover a number of plausible targets of co-selection in *S*. *pyogenes* linked to RNA pseudouridine synthases. Our method significantly enhances the future potential of epistasis analysis for systems biology, and can complement genome-wide association studies as a means of formulating hypotheses for targeted experimental work.

## Introduction

The study of co-evolution in recombining populations of bacteria has been limited by the scale and polymorphisms present in population samples for which whole genome sequences are available. Even the most recent population genomic studies of bacterial pathogens have been constrained in this respect, such as focusing on a particular genotype[1–3], biasing sampling towards particular clinical outcomes[4–6], or surveying organisms in which limited genetic diversity and strong linkage disequilibrium (LD) mask the signals of shared selection pressures[7,8]. For whole genome-scale modeling of co-evolution, sampling should preferentially span the entirety of a diverse, recombining species in an unbiased manner.

The first organism satisfying all the above-mentioned desiderata is *Streptococcus pneumoniae* (the pneumococcus), for which over 3,000 genome sequences from a well-defined, limited study population were recently published[9]. As the pneumococcus is an obligate nasopharyngeal commensal and pathogen, the bacterial population was evenly sampled through a structured survey of the hosts. The diverse multi-strain population structure, coupled with the naturally transformable nature of *S*. *pneumoniae*, results in low LD across the genome. Hence this set of pneumococci can be considered as an ideal set for detecting genes that evolve under shared selection pressure. In contrast, we also investigate how well a genome-wide analysis of co-evolution can perform when the sampling is done from the opposite end of the genetic variation spectrum by considering 3,442 genomes of the M1 lineage of *S*. *pyogenes*, which has contributed significantly to the global epidemic of group A Streptococcus infections during the past three decades[3]. In this case recent expansion from a single progenitor has generated a clonal population that has experienced a minimal amount of homologous recombination.

Analyzing sets of co-evolving polymorphisms is a powerful means of identifying sites that interact directly, through protein-protein contacts, and indirectly, through epistatic interactions that affect the same phenotype. The former type of selection pressure has previously been studied on the scale of individual proteins. It has been known for more than 20 years that the correlations of amino acids in two columns in a multiple sequence alignment (MSA), contain exploitable information and provide a non-trivial predictor of protein tertiary structure spatial proximity[10,11]. Detection of co-evolving mutations in genomes is in the statistical sense analogous to this structural prediction problem, as both phenomena can arise as a consequence of joint selection pressures. The latest advances in computational structural biology have shown that by changing the modeling framework from correlations to high-dimensional model learning one can improve protein contact predictions significantly, an approach generally referred to as *direct coupling analysis* (DCA)[12–15]. Furthermore, including considerations of epistatic interactions between sites has recently been shown to significantly improve the mapping between genotype and phenotype for a beta lactamase protein[16].

Co-evolving sites do not necessarily directly interact. Rather, changes at distinct sites may represent selection for a particular phenotype determined by multiple polymorphic loci. However, the complexity of the possible set of interactions has mostly limited previous analyses of epistasis to viral datasets of limited diversity; nevertheless, these studies have shown epistasis to be an important factor in evolution. An application of a phylogenetically-informed method to influenza subtypes H1N1 and H3N2 identified patterns of substitutions associated with the emergence of resistance to oseltamivir[17], and many sites were found to be undergoing coordinated evolution within the hepatitis C virus[18]. However, the non-linear expansion in the number of interactions as the genome length and diversity increase has hampered the application of such methods to the study of bacterial populations. In recent work, pairwise statistical correlation analysis was demonstrated to successfully reveal certain types of co-evolutionary patterns across the genome for 51 *Vibrio parahaemolyticus* isolates[19]. While this approach appears promising given the way in which linkage disequilibrium is handled in it, pairwise analyses of association are in general subject to Simpson’s paradox which may cause difficulties in separating direct and indirect links between variables[20–22]. Furthermore, the necessity of correcting for a quadratically increasing number of multiple hypothesis tests reduces the statistical power to detect the true positive associations. A model-based approach to estimating the strength of co-evolution between genome sites is therefore preferential to correlation based analysis, as has been clearly demonstrated earlier in the context of protein evolution[12–15].

Here we demonstrate a new method for the identification of statistically significantly co-evolving polymorphisms from bacterial genome sequence alignments named genomeDCA (freely available at https://github.com/mskwark/genomeDCA). By considering the evolution of polymorphic sites simultaneously and using the inference tools for regularized statistical model learning one avoids both the problems that drive high levels of false positives and negatives when the number of pairwise interactions grows. The method introduced here offers a powerful complementary approach to traditional GWAS analyses[23] for the explorative discovery of polymorphisms potentially underlying phenotypes that have an unknown genetic basis in the emerging era of massive population sequencing for bacteria.

## Results

### Genome-wide identification of coupled loci for the pneumococcus

**Fig 1:**
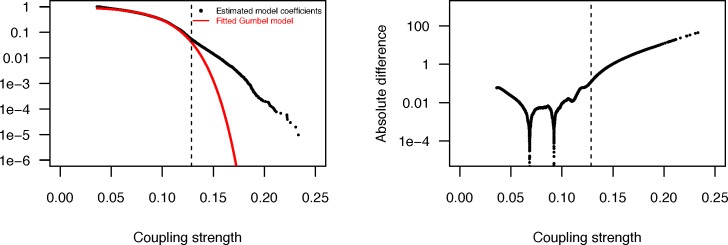
Divergence between theoretical and empirical distributions of coupling strengths between sites. Left panel (A) shows the two distributions such that the vertical axis corresponds to the log10 probability of a coupling coefficient exceeding the value of the curve on the horizontal axis. The dashed vertical line depicts the significance threshold; 5199 out of 102,551 couplings exceed the threshold. Right panel (B) displays the absolute difference between the fitted cumulative Gumbel distribution and the empirical cumulative distribution (on log10-scale) as a function of the coupling strength. The dashed vertical line marks the smallest coupling (0.129) which has a difference of more than six standard deviations among the first 50,000 empirical-Gumbel differences.

**Fig 2:**
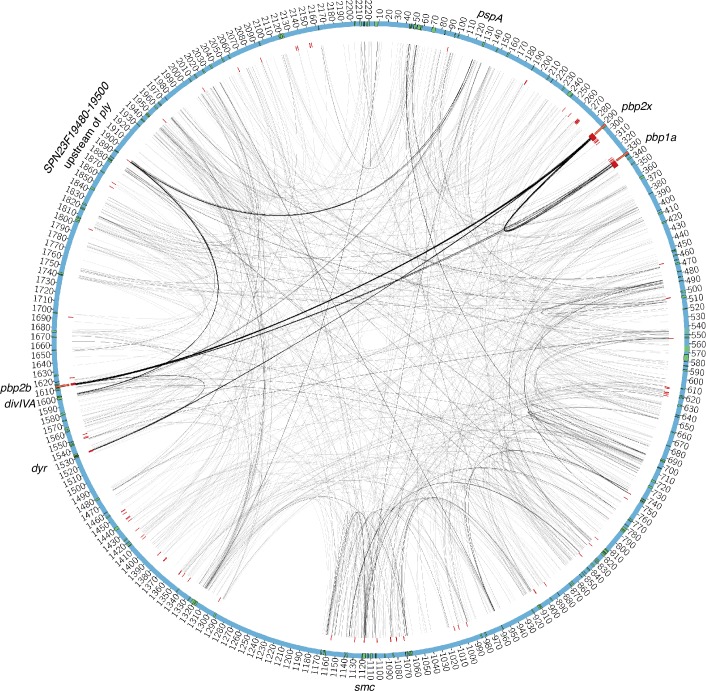
The 5199 significant couplings shown by lines connecting genomic positions which are indexed in kilobases by the running numbering. The thickness of lines is proportional to the number of linked positions within the corresponding chromosomal elements. The red markers show the positions of sites identified in an earlier GWAS study of resistance determining variation in the pneumococcal genomes. The green markers indicate locations of protein coding sequences where significant couplings are present. Gene annotations shown outside the circle are centered at the positions of the corresponding genes.

Analysis of coupled loci for the pneumococcus is derived from a whole genome alignment generated from short-read data for 3,156 systematically-sampled pneumococcal isolates [9] each aligned to the reference sequence of *S*. *pneumoniae* ATCC 700669 [24]. Filtering this alignment for biallelic loci at which the minor allele frequency was >1% and >85% of isolates had a base called identified 81,560 polymorphic sites. Of these, 88.3% were within protein coding sequences (CDSs), a slight enrichment relative to the 87.2% of the *S*. *pneumoniae* ATCC 700669 reference sequence annotated as CDSs. Following the genome-wide linkage analysis (see Methods), estimates of association strength were retained from 102,551 couplings (S1 Table). A Gumbel distribution fitted to this sample (Fig 1; μ = 0.096 and β = 0.028) significantly diverged from the empirical data above a coupling strength of 0.129. The 5,199 couplings (S2 Table) exceeding this threshold were considered as putative epistatic interactions; these affected 1,936 sites, 89.0% of which were within CDSs. As closely proximal sites were excluded from this analysis, these coupled sites had a mean separation of 587.4 kb, with only two sites separated by less than five kilobases (Fig 2). Hence these associations are unlikely to be artifacts of genetic linkage.

### Strong epistatic links between penicillin-binding proteins

**Fig 3:**
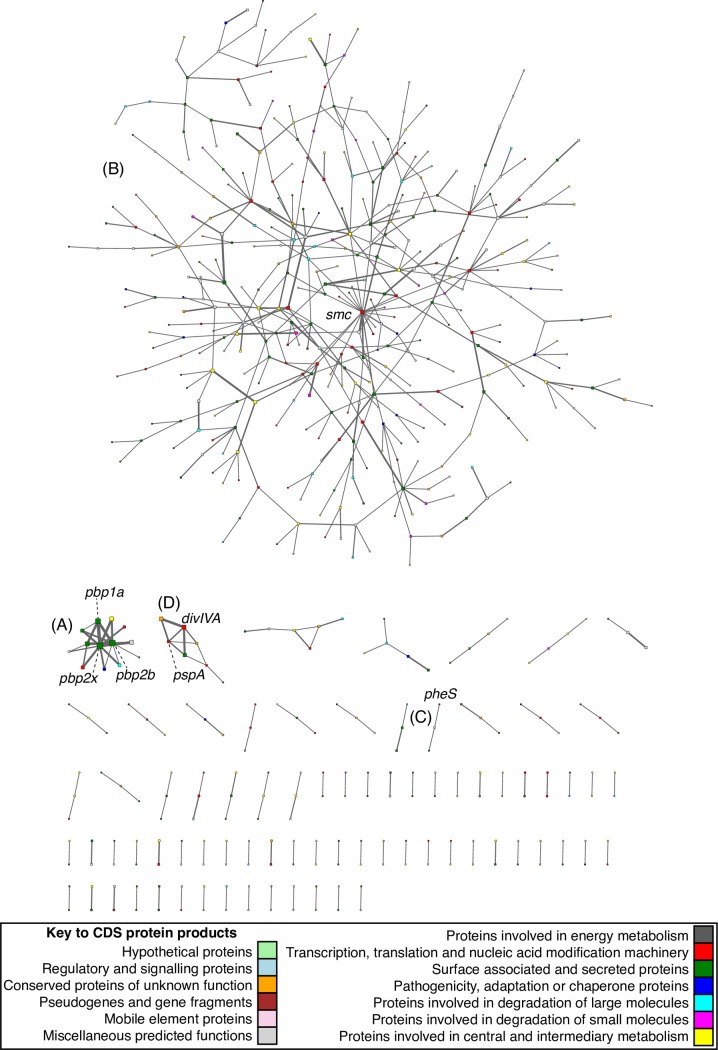
Network of coupled protein coding sequences. This undirected network shows all significant couplings between protein coding sequences (CDSs). Each node is a CDS, colored according to its functional annotation, and scaled according to the logarithm of the number of significant coupled loci it contained. Edges are weighted according to the logarithm of the number of significant coupled loci linking two CDSs. (A) Network component containing the genes *pbp2x*, *pbp1a* and *pbp2b*. (B) Network component containing the *smc* gene. (C) Network component containing the tRNA synthetase gene *pheS* and a coding sequence for another putative tRNA-binding protein. (D) Network component containing the genes for *pspA* and *divIVA*.

The putative epistatic interactions identified by genomeDCA were used to generate a network (Fig 3). The nodes, each corresponding to a CDS, were colored according to function and scaled according to the number of epistatic links with which they were associated. The edges were weighted according to the number of interactions between CDS pairs. Most of the annotated functional categories were represented in the network, with the notable exception of mobile genetic element genes. This absence was almost entirely the consequence of the lack of informative sites in these regions of the genome, owing to their high variability across the population. By contrast, the functional category most over-represented in the dataset was surface-associated proteins. Although previous work has suggested immune selection might drive epistasis between antigens [25], in fact this enrichment was entirely the consequence of selection for antibiotic resistance. Of the 4,617 links represented in this network, 3,578 (77.5%) involved 175 sites found in one of three genes encoding penicillin-binding proteins (PBPs; Fig 3A): SPN23F03080 (*pbp2x*), SPN23F03410 (*pbp1a*) and SPN23F16740 (*pbp2b*). These PBPs have been experimentally demonstrated to be the major determinants of resistance to beta lactams [26], with changes to each individual protein reducing its affinity for beta lactam antibiotics while retaining its the ability to bind its natural substrates[27,28]. This link between genotype and phenotype was also identified by a genome-wide association study (GWAS) using this dataset[29]. Of the 858 sites found to be significantly associated with beta lactam resistance by this GWAS, 403 were within these three coding sequences for PBPs; of these, 216 met the criteria to be analysed in this study. These corresponded to 161 of the 175 sites identified within the same genes by this analysis, representing a highly significant overlap with those sites significantly associated with beta lactam resistance by the GWAS (Fisher exact test, OR = 110.2, 95% confidence interval = 58.85–221.83, *p* < 2.2x10^-16^). Hence the couplings between sites in these genes represent a set of changes that distinguish pneumococci with differing sensitivities to beta lactam antibiotics. This was in line with the distribution of these alleles across the population (S1 Fig), with particular alleles at the coupled sites found in multiple penicillin-insensitive lineages. This also confirmed that none of the identified associations correlated with the expansion of a single clone.

**Fig 4:**
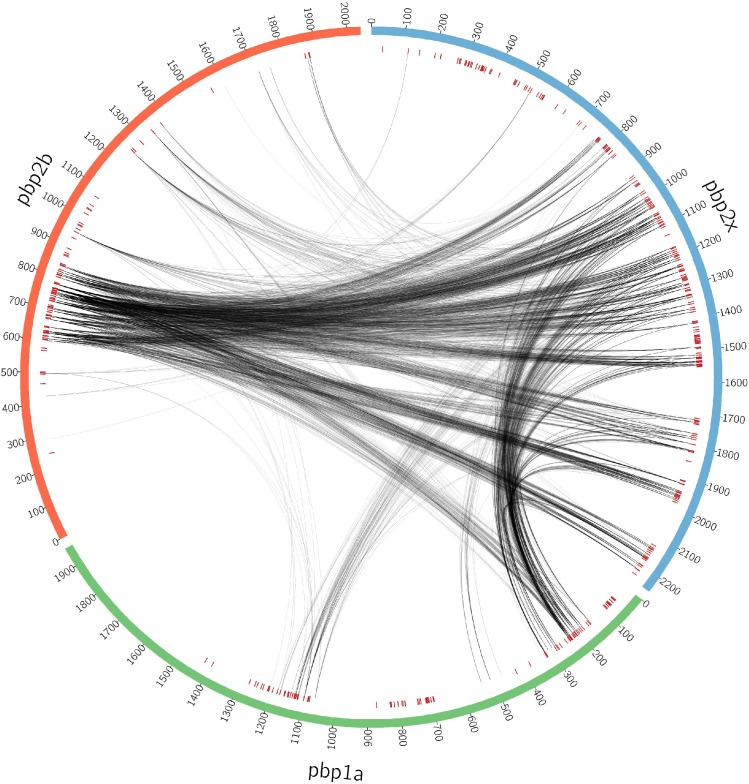
Distribution of couplings between sites in different PBPs. The red markers are defined as in Fig 2.

Changes in these genes are strongly epistatic, as alterations of all three PBPs are necessary for pneumococci to develop non-susceptibility to a broad range of beta lactam antibiotics. Consequently, the emergence of multidrug-resistant pneumococci is associated with genetic changes that alter all three of these enzymes over very short evolutionary timescales [30–32]. Another factor that might underlie both the concerted changes at all three loci, rather than a gradual emergence of resistance, as well as the non-uniform distribution of coupled sites across these genes (Fig 4) is the potential for alterations in only one protein disrupting direct protein-protein interactions. As these proteins perform similar functions on the same substrate, and are all co-localised to the cell membrane, it has been hypothesised that they function as constituents of a multi-enzyme complex[33]. Evidence from co-immunoprecipitation and crosslinking experimental work has supported this idea[34,35] and hence the distribution of coupled sites between the PBPs was investigated in greater detail.

### Structural distribution of coupled sites in PBPs

Couplings were identified between all three PBPs, although almost 95% involved *pbp2x*. This might reflect that *pbp2x* has to be altered for resistance to both penicillins and cephalosporins, whereas modifications of *pbp1a* are important primarily for cephalosporin resistance [36], and modifications to *pbp2b* are important primarily for penicillin resistance [37]. Alternatively, if these proteins do interact, these data suggest *pbp2x* would be central to any potential protein-protein interactions. The coupled sites are distributed broadly across *pbp2x*, with the exception of the PBP dimerization domain, despite the identification of sites within this region by GWAS.

**Fig 5:**
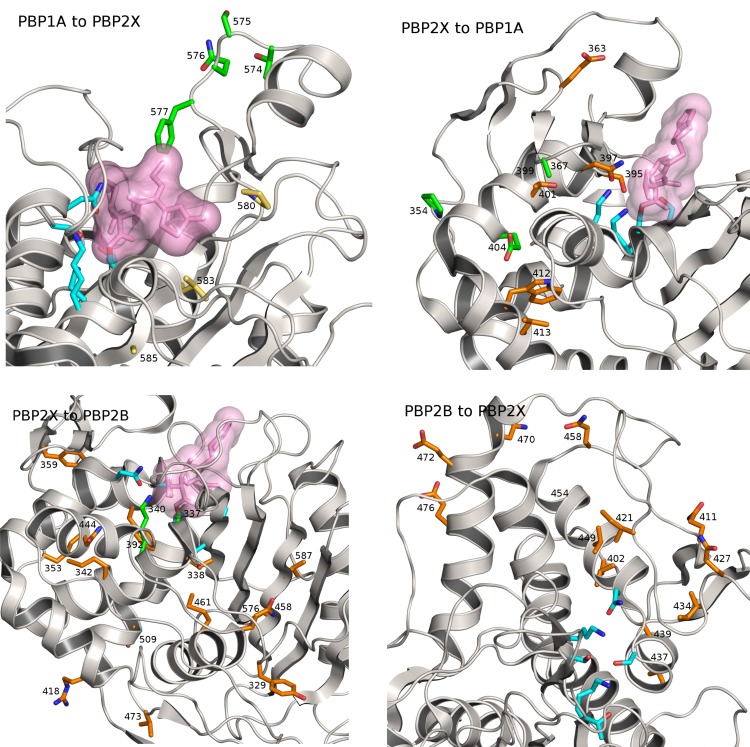
Structural models of *pbp1a*, *pbp2x*, *pbp2b* with the 100 strongest couplings listed in S3 Table indicated. The figures show the transpeptidase domains of each PBP with catalytic/active site residues shown in cyan and coupled positions as sticks with other colors. Active site bound antibiotic/inhibitor is rendered as a space-filling volume when present in the crystal structure. Panels A-D depict: *pbp1a* with couplings to *pbp2x*, green colored residues are coupled with green residues in panel B; orange colored residues in B are coupled with both green and yellow residues in A (A), *pbp2x* with couplings to *pbp1a* (B), *pbp2x* with couplings to *pbp2b* in orange (C), *pbp2b* with couplings to *pbp2x* in orange (D).

Co-evolving sites related to *pbp1a* are more narrowly distributed and involve stronger interactions with *pbp2x* than with *pbp2b* (Fig 4, S2 Table). The links between the proteins PBP1A and PBP2X are distributed between two domains and a further structural analysis (Fig 5, S3 Table) showed how the identified positions in *pbp1a* with the strongest couplings were located in strand β-4 and the loop connecting strands β-3 and β-4, near the transpeptidase active site, but not overlapping with the conserved catalytic residues. The counterpositions in *pbp2x* showed a spatially less focused pattern with linked positions near the active site, including the typically conserved active site residues Ser395 and Asn397, as well as links to positions not in direct spatial proximity of the active site, but structurally linked to that region through helical secondary structure elements. Alterations in the active site surroundings can interfere with inhibitor binding with only minor effect to catalytic function as was evident for substitutions in the identified β-3/β-4 loop region residues 574–577 which were strongly linked to beta-lactam resistance[38]. Resistant strain *pbp1a* likewise showed amino acid substitutions at the identified positions 583 and 585 in strand β-4 in comparison to a beta-lactam susceptible strain[38] (resistant strain PDB code 2V2F). Position 580 at the N-terminal end of β-4 (typically a proline) in *pbp1a* was linked to position 363 in *pbp2x*, which is part of an ionic interaction (Glu363 to Arg372) present in all current crystal structures of *S*. *pneumoniae pbp2x* (see Methods for details) and may play a structural role by stabilising the α-2/α-4 loop region proximal to the *pbp2x* active site. Residues at PBP2X positions 401, 404, 412 and 413 are all buried within the protein, but are connected to active site residues Asn397 and Ser395 via helix α-5. It is possible that these positions are implicated in active-site shaping as well. For *pbp2b*, structural mapping of the top ranking co-evolved sites revealed two major groupings: positions in the α-2/α-4 loop region that, similarly to the *pbp1a* case, partially cover the active site, and positions that, similarly to the *pbp2x* side of *pbp2x* –*pbp1a* couplings, were spatially more distant but structurally linked to the active site. As in *pbp1a*, observed flexibility in the α-2/α-4 loop region proximal to the active site points to a potential role in antibiotic resistance of this structural feature[39].

### Associations with other resistance phenotypes

The PBPs are confined to a single network component that contains ten other proteins. Seven of these are found in close proximity to the three PBPs, and likely represent sequences altered when resistance-associated alleles of the PBP genes were acquired through transformation events, which often span tens of kilobases [30,32]. However, it is also possible these could play a role in ‘compensating’ for deleterious side-effects of the changes in the PBP proteins. One of these CDSs proximal to a penicillin-binding protein gene is *mraY*, directly downstream of *pbp2x* and encoding a phospho-N-acetylmuramoyl-pentapeptide-transferase also involved in cell wall biogenesis (S2 Table, Fig 3). It was previously predicted that mutations in this transferase associated with beta lactam resistance could represent compensatory changes ameliorating the costs of evolving beta lactam resistance[29]. Another CDS, *gpsB*, is shortly upstream of *pbp1a* and encodes a paralogue of DivIVA that also plays an important role in peptidoglycan metabolism [40].

The three proteins in the network component that were not proximal to a PBP-encoding CDS were *dyr* (also known as *folA* or *dhfR*), encoding dihydrofolate reductase, and three nearby genes (S2 Table, Fig 3). Mutations in the *dyr* gene cause resistance to trimethoprim [41]. The earlier GWAS study[29] found a significant association between both *dyr* and *folP* with beta lactam resistance, despite no functional link to such a phenotype, nor any likely reason why they would directly interact with PBPs. Hence the detected interaction between *dyr* and the *pbp* genes is most likely explained by the co-selection for resistances that have accumulated in the same genetic background, resulting in the multi-drug resistant genotypes observed to have emerged over recent decades[42].

### Couplings between core genome proteins

To identify other functional roles that might underlie the distinct sets of couplings represented in Fig 3, a gene ontology (GO) analysis was performed for each network component containing more than two nodes. This identified five significant signals, including that for penicillin-binding associated with the previously described component (GO:0008658, Fisher’s exact test, OR = 337.3, 95% confidence interval = 25.40–15878.4, *p* = 0.00048 after Benjamini-Hochberg correction). However, the strongest association was that of the largest network component, containing 384 CDSs (Fig 3B), with ATP binding activity (GO: 0005524, Fisher’s exact test, OR = 2.89, 95% confidence interval = 2.01–4.18, *p* = 8.75x10^-7^). Other GO terms significantly associated with this component were GO:0005737, corresponding to cytosolic localisation (Fisher exact test, OR = 3.44, 95% confidence interval = 2.10–5.62, *p* = 0.0001 after Benjamini-Hochberg correction), and GO:0016021, corresponding to integral membrane proteins (Fisher exact test, OR = 2.32, 95% confidence interval = 1.45–3.66, *p* = 0.043 after Benjamini-Hochberg correction), which have cytosolic segments, despite being surface associated. These associations partly reflect the preponderance of cytosolic ATP-hydrolysing tRNA synthetases, of which enzymes for the processing of eleven amino acids were present among these CDSs, and membrane-associated ATP-hydrolysing ABC transporters. The most highly connected node in the component, linking to 22 other CDSs, was another ATPase. SPN23F11420, encoded the Smc protein, is critical in organizing the chromosome and forms the basis of a multi-protein complex in both prokaryotes and eukaryotes [43]. Hence this large diverse set of coupled CDSs included many components of the essential cytosolic machinery, the interactions of which are critical to the basic functioning of the cell.

The fourth significant enrichment of GO terms also involved tRNAs (GO:0000049—tRNA binding; Fisher exact test, OR = 869.1, 95% confidence interval = 36.83–4.50x10^15^, *p* = 0.00048 after Benjamini-Hochberg correction), which applied to a component containing three nodes (Fig 3C). One corresponded to *pheS*, a phenylalanyl tRNA synthetase, while the other was SPN23F19340, annotated as encoding a tRNA binding protein of unknown function. Attempting to identify a more specific functional prediction using the CDD database[44] we found this protein possessed a “tRNA_bind_bactPheRS” domain, specifically involved in processing phenylalanyl-tRNAs, and only otherwise found in PheT, which directly interacts with PheS in the phenylalanyl-tRNA synthetase. Hence this coupling may represent a previously unexpected direct protein-protein interaction.

The CDS directly downstream of *pheS*, encoding a putative membrane-associated nuclease (SPN23F05260), was coupled to a tRNA methyltransferase adjacent to *pspA* in a separate network component (Fig 3D). The *pspA* gene, encoding a surface-associated protein involved in pathogenesis and immune evasion, was itself present in the same network component, and engaged in some of the strongest coupling interactions in the dataset. These linked to the *divIVA*, encoding a cell morphogenesis regulator [40], and three CDSs upstream of *ply*, encoding the major pneumococcal toxin pneumolysin[45] which, like *pspA*, is critically important in pneumococcal virulence and upregulated during infection[46]. These three *ply*-associated CDSs (SPN23F19480-SPN23F19500) encode proteins likely to play a role in localising or transporting pneumolysin from the cytosol into the cell wall[45]. Hence these coupling links could be the consequence of these virulence proteins engaging in interactions at the surface of the cell.

Interactions were also detected between CDSs and non-CDS sequence. The 213 non-CDS coupled sites were enriched in non-coding RNAs (Fisher exact test, OR = 1.92, 95% confidence interval = 0.963–3.48, *p* = 0.041), suggesting they may represent functional links between RNA and proteins. However, the non-coding RNAs involved were riboswitches in 5’ untranslated regions. When a bipartite network was constructed that displayed couplings between CDSs and upstream non-CDS regions (S2 Fig), the network components mirrored those in Fig 3. This suggested the links represented sequence proximal, and therefore linked, to CDSs that were coupled, rather than epistatic interactions involving direct protein-DNA interactions. Correspondingly, neither DNA binding (GO: 0003677) nor RNA binding (GO:0003723) were enriched in this network. Similarly, there was no enrichment of coupled sites in non-coding regions between divergently transcribed CDSs, which should be enriched for regulatory elements (Fisher test, OR = 0.48, 95% confidence interval = 0.283–0.757, *p* = 0.00074).

### Genome-wide identification of coupled loci for the group A *Streptococcus* M1 population

**Fig 6:**
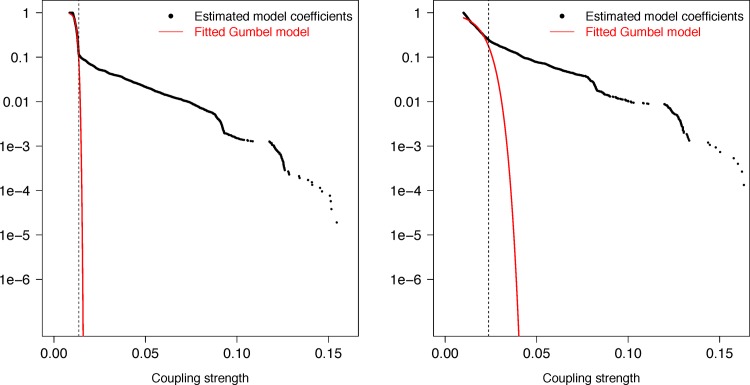
Divergence between theoretical and empirical distributions of coupling strengths between sites for *S*. *pyogenes*, defined as in Fig 1. Left panel shows the distributions for the 324 locus data set and right panel for the 5078 locus data set.

**Fig 7:**
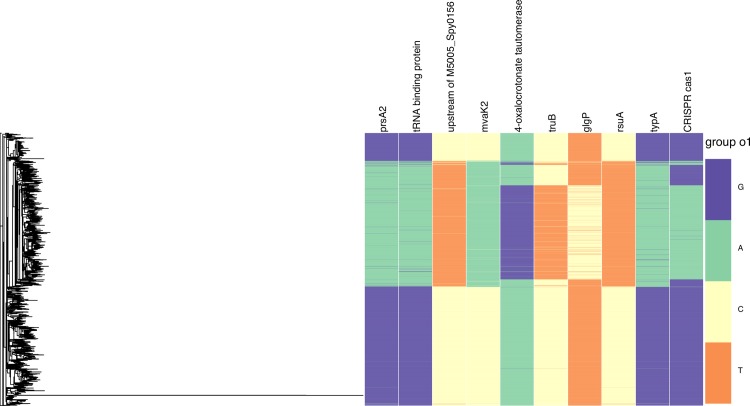
Phylogeny of the M1 lineage and the distribution of minor/major alleles in the SNP loci involved in the 20 most highly ranked significant couplings.

The whole genome alignment used in this analysis consists of short-read data for 3,442 isolates belonging to the contemporary M1 lineage of *S*. *pyogenes*, which has contributed significantly to the global epidemic of group A *Streptococcus* infections during the past three decades[3]. Filtering this alignment for polymorphic sites at which the minor allele frequency was >1% resulted in 324 SNP loci (coupling estimates in S4 Table). To investigate the effect of including SNPs with more rare minor alleles we also performed an analysis without such MAF based filtering. However, uninformative polymorphic sites where the minor allele was private to a single genome only were still excluded in this analysis, leaving 5078 SNPs out of the total of 12400 SNPs present in the alignment (coupling estimates in S6 Table, http://dx.doi.org/10.5061/dryad.gd14g). Similar to the pneumococcus analysis, we employed the Gumbel model to determine a threshold for significant couplings (Fig 6). For the two sets of loci there are 5952 and 1840 couplings exceeding the thresholds 0.014 and 0.023, respectively. Inclusion of loci with rare minor alleles (MAF < 1%) did not impact rankings of the strongest couplings found in the smaller locus set. None of the loci with the rare minor alleles were among the 2000 top ranking couplings, while 5.3% of couplings ranking between 2000–3000 involved any such loci. However, their fraction was approximately 30% among couplings ranking between 3000–5000, which indicates that relatively rare minor alleles may also lead to moderate levels of coupling signals when they are tightly linked as is the case for M1 data. Nevertheless, the vast majority of the loci with MAF < 1% were included in couplings very close to zero (S5 Fig). Since the M1 lineage stems from a recent expansion from a single progenitor cell, there has been on an evolutionary timescale relatively little opportunity for recombination to disrupt the clonal frame resulting from this genome-wide population sweep, consequently the SNP loci are relatively tightly linked across the whole chromosome. To better enable an inspection of the biological meaning of the significant 5952 couplings, we ranked them using successively the following three criteria: 1) size of the coupling coefficient (rounded off to two decimal points), 2) percentage of isolates where both SNP loci involved in a coupling had the minor allele, 3) minimum of the average genome-wide Hamming distances of isolates carrying the minor alleles at the two loci, respectively. Large values of the latter two criteria emphasize cases where both minor alleles at a strongly coupled pair of loci are simultaneously widely distributed across the population, i.e. maximize their phylogenetic spread. The loci included in the twenty highest ranking couplings are visualized together with the maximum likelihood phylogeny in Fig 7.

The overall pattern of couplings between *S*. *pyogenes* genes lacked a pattern akin to the very strongly linked penicillin-binding proteins observed in *S*. *pneumoniae*. However, there were some similarities evident in comparison of the results obtained from the two population genome data sets. Of the twenty coupling interactions ranked most highly according to the above stated criteria, eight involved two RNA pseudouridine synthases, despite only eight such enzymes being annotated in the chromosome. The 16S rRNA U516 pseudouridylate synthase gene *rsuA* (M5005_Spy1092) accounted for five couplings, one of which linked to a CDS (M5005_Spy0101) directly orthologous with the tRNA binding protein identified in the analysis of *S*. *pneumoniae* (SPN23F19340). Other linked genes involved in nucleotide metabolism were the *prsA2* ribose-phosphate pyrophosphokinase (M5005_Spy0018) and the GTP-binding transcription factor *typA* (M5005_Spy1255). The tRNA pseudouridine synthase gene *truB* accounted for a further three couplings, one of which was to the Cas1 nuclease-encoding CDS M5005_Spy1286, part of the RNA-dependent *S*. *pyogenes* CRISPR2 system. These polymorphisms were homoplasic, as indicated by the phylogeny in Fig 7, demonstrating the pairings have arisen on multiple occasions, and do not simply reflect common descent.

### Effect of the population structure correction

The smaller dimensionality of the M1 data set allowed us to systematically vary the level of population structure correction in the pseudolikelihood inference and study its effect on the estimated couplings. Since the inference was computationally expensive for the pneumococcus data, only the default level of correction (0.90) was used for all the resampling based analyses. S6 Fig shows that the ranking of the few thousand top couplings is stable across threshold levels 0.75–0.90, whereas the lower thresholds pull more strongly some of the uncorrected couplings ranked between 4000–10000 towards the lowest ranking (around 50000). In contrast, the threshold 0.95 appears to be too high since the ranking becomes less stable and some of the weaker signals are given too much emphasis.

## Discussion

Natural selection continuously shapes genetic variation in bacterial populations, acting to purge sequence variation that is deleterious and to maintain variation that is beneficial. Laboratory experiments provide the gold-standard method for establishing underlying mechanisms among observed variable sites. However, such experiments also necessitate the definition of a measurable phenotype, which may be a daunting task for many complex traits relevant for survival and proliferation of bacterial strains. The exponentially increasing size of the genome sequence databases provide a valuable resource for generation of hypotheses for experimental work. In eukaryotes, GWAS methods have been used for more than a decade to probe for DNA variation that is non-randomly allied with phenotypic differences indicative of a possible causal genotype-phenotype relationship [47,48]. In bacteria, use of GWAS for this purpose is of much more recent origin but has been demonstrated to hold a considerable promise in the light of more densely sampled populations[23,29,49–52]. However, GWAS is not the only way in which wealth of bacterial sequence information has been proposed to be used to gauge which genes could potentially be targets of positive selection and to generate hypotheses for experimental work. For example, Li et al. screened genome sequences of closely related pairs of isolates in a densely sampled pneumococcal population which would differ at particular genes of interest to provide candidate targets for phenotypic tests[53].

By leveraging from the most recent advances in computational protein structure prediction and statistical machine learning, we have been able to introduce a method that promises to complement the popular GWAS approach for understanding how polymorphisms affect phenotypic variation. This work identified many different coupled sites across the genome, which network analysis revealed to define separate clusters of genes involved in resistance, virulence, and core cell functions. Our study represents the first attempt to use statistical modeling to fully exploit large-scale bacterial population genomics to identify patterns of co-evolution in sequence variation.

As illustrated by the genomic data from the contemporary M1 lineage, when a population has experienced a very limited amount of homologous recombination, it will be more challenging to separate couplings related to LD from those that are under shared co-evolutionary pressure. Our further analysis ranking pairs of SNP loci by the degree of phylogenetic spread of the minor alleles and their matching at both loci in addition to the strength of the coupling, illustrates that it may be possible to deduce biologically relevant signals even under such circumstances. Further research on different ways to post-process the coupling estimates to more precisely reflect different evolutionary scenarios is therefore warranted.

Our approach provides a comprehensive genome-wide view of epistasis, which is both quantitatively and qualitatively different from studies of coupled evolution between functionally-related genes or proteins. Results applying to single pathways or functions, such as the penicillin-binding proteins, likely reflect strong selection for functionally important interactions. Many such interactions are likely to exist throughout the pneumococcal genome, particularly related to central and secondary metabolism, but will not be detected in this study for two reasons. Firstly, the operonic organisation of coding sequences means such sites will often be in linkage disequilibrium with one another, and therefore excluded from this analysis on the basis of proximity within the chromosome. Secondly, strong selection against mismatched alleles at interacting sites would be expected to limit the maintenance of diversity at these sites, meaning minor allele frequencies above the threshold required in this study will not be attained. This genomeDCA method instead highlights interactions important in shaping the evolution of a population, such as the diversification of the pneumococcal penicillin-binding proteins. These unlinked genes have been under strong selection to decrease their affinity for beta lactams in some isolates, yet still co-existing with sensitive isolates, resulting in the genetic variation necessary for both coupling analyses and GWAS.

The biological interpretation of the many other coupled sites should account for the possibility of being driven by the avoidance of deleterious non-productive interactions, necessary for the maintenance of binding specificity between interacting proteins. These can only be detected through a whole genome approach, providing a previously unattainable insight on those interactions that cannot be anticipated from *a priori* functional analyses. Such non-productive interactions are, by definition, more numerous than the specific interactions between functionally related proteins. Furthermore, each individual interaction is likely to be under weaker selection compared with specific functional interactions, and therefore detectable levels of variation are more likely to be maintained at the relevant polymorphic sites across the population. Therefore not all couplings detected in this work will represent functional links; some will instead provide a new type of data about the ‘noise’ inherent in an imperfectly specific complex biological system. This type of interaction is very difficult to detect in a high-throughput manner by any other methodology, and may well play a previously underappreciated role in bacterial evolution.

Importantly, as we have demonstrated, it is not necessary to specify the relevant phenotypes *a priori* for this approach to work successfully. Therefore a single analysis with this method may simultaneously reveal co-selected sites for many different traits, and provide an indication of their relative influence on evolutionary patterns, as illustrated by the results on the pneumococcus. The selective pressure for at least some of the population to develop beta lactam insensitivity clearly has a substantially greater influence on the co-evolution of unlinked sites than any other loci in this population. Since our approach is widely applicable to data generated by bacterial population genomic studies, it has considerable potential to identify important targets for subsequent experimental work designed to gain system level understanding about the evolution and function of bacteria.

## Materials and methods

### Genome data

The 3,156 genomes of the pneumococcus used in our study are obtained from the study by Chewapreecha et al[9], including 71 additional genomes not presented in their original article. A list of the accession numbers of all the 3,156 genomes is provided in the Supplementary materials (S5 Table) and the multiple sequence alignment is available from the Dryad Digital Repository: http://dx.doi.org/10.5061/dryad.gd14g. We used a genome alignment produced as in Chewapreecha et al[9] of the total length 2,221,305 bp, in which 388,755 SNP loci were present, out of which 134,037 loci had a minor allele frequency (MAF) of at least 0.01. Out of these we selected 81,560 loci that had coverage of at least 84.1%, i.e. not more than 500 genomes with a gap/unresolved base pair at the considered sequence position. As the analysis focused solely on the biallelic loci, the observed nucleotides have been replaced, so that the entire alignment was composed of only three letters (two representing observed allele (major/minor) and one gap/unobserved allele). This was done in the interest of reducing the number of parameters of learnt models approximately 3-fold.

The 3,442 genomes of the *S*. *pyogenes* were obtained from the study by Nasser et al. [3], representing the post-resurgence epidemic strains of the original article, that is, the contemporary lineage. The multiple sequence alignment is available from the Dryad Digital Repository: http://dx.doi.org/10.5061/dryad.gd14g. We used the genome alignment produced in Nasser et al. with the total length of 1,838,562 bp in which 12400 SNP loci were present for the post-epidemic strains. Maximum likelihood phylogeny was estimated with RAxML[54] using the 12400 SNPs and the GTR+Gamma model with 100 bootstraps. The same filtering criteria were used as for the pneumococcus, which resulted in 324 SNP loci being included in the genomeDCA analysis. To visualize the phylogeny together with the allele distributions observed at a subset of the SNP loci, we used the Phandango software available at http://jameshadfield.github.io/phandango/. In addition, analysis was also performed without MAF based filtering, such that only SNP loci where the minor allele was private to a single genome were excluded. This analysis included 5078 out of the 12400 SNPs present in the alignment.

### DCA and regularized model learning

Direct Coupling Analysis (DCA) was introduced as a method to predict residue-residue contacts in protein structures from multiple sequence alignments (MSAs) of many homologous proteins[12,55]. A general review about the use of DCA in protein contact prediction has been recently made by de Juan et al.[56] The essence of DCA is to use the data to learn a probabilistic model in an exponential family referred to as a Potts model, and to use model parameters to characterize the data. Potts models are generalizations of the well-known Ising model[57], where each variable can take *q* different values (*q = 2* for the Ising model); for the contact prediction problem *q* equals *21* corresponding to the twenty normally occurring amino acids and a gap state in an MSA. Potts models and Ising models contain linear (one-variable) and quadratic (two-variable) terms; in DCA the quadratic terms, also referred to as *couplings* or *interactions*, are used to characterize the data. For completeness we give a mathematical definition of Potts models in Supplementary Information, where we also give further details on how these models are parametrized.

In this work we use the MSA built on loci of variation in the genome sequences, filtered such that at each locus in one sequence we only have a major allele, a minor allele, or the locus can be missing. A sequence is thus coded as a string of symbols major/minor/gap, and we use the table of such strings to learn a Potts model with *q* = 3 states. A coupling between a pair of loci can be parametrized by a 3x3 parameter matrix. The parametric dimensionality consequently grows by the number of loci squared, i.e. is here on the order of 10^10^ for the pneumococcus data, whereas the number of observations is on the order of 10^3^. Models are therefore regularized as discussed in Ekeberg et al[58].

A central aspect of the DCA procedure is that only a set of strongest predicted interactions are retained[12,55]. Indeed, as the number of parameters of these models in practical use is much larger than the sample size, a selection is necessary. For the contact prediction problem the number of retained predictions has often been taken to be about the length of the protein[14,59]. Here we have determined instead a threshold for significant interactions using the statistical theory of extreme value distributions, as explained below.

The final central aspect of DCA is the actual learning procedure, or *inference*. Exact solution of maximum likelihood (ML) inference of general Ising and Potts models is a computationally hard problem and not feasible for the instances of interest; in statistics such likelihood functions are often referred to as *intractable*. Several approximate learning schemes have therefore been developed[12,55,58,60–64]. DCA for protein contact prediction has also been combined with other methods[59,65]. We have in this work used pseudolikelihood inference as described below.

Deriving the meaning of inferred Potts model parameters in a highly under-sampled situation is not a simple mathematical problem, and no agreement has so far been reached in the DCA literature[66,67]. For instance, the numerical values of inferred parameters depend on the regularization, such that large regularization yields small inferred parameter values. As in most DCA studies we have in this work relied on the empirical observation, discussed e.g. in Ekeberg et al.[62], that the *order* of the largest inferred parameters is only weakly dependent on regularization. The subsequent analysis is then based on the identity of these largest predictions, while the corresponding numerical values are not used further. For a recent theoretical discussion of the performance of DCA for contact prediction compared to faithfully reconstructing the full probability distribution over sequences, see Jacquin et al.[68]

### Pseudolikelihood inference and parameter scoring

Pseudolikelihood was originally introduced in the early 1970’s to enable estimation of parameters in spatial statistical models with intractable likelihood functions[69,70]. This inference technique has experienced a strong revival in the recent years for high-dimensional applications where the number of possible model parameters greatly exceeds the number of observations, known as the ‘small *n*, large *p*’ problem[71]. In particular, pseudolikelihood provides consistent estimators of the model parameters unlike the competing variational inference methods[72]. The outcome of the inference is a set of matrices *J*_*ij*_ describing the interactions between loci *i* and *j*. We score these numbers by their Frobenius norms |*J*_*ij*_|, which we refer to as *interaction strengths*, but without the Average Product Correction (APC) which has become the most common choice in the protein contact prediction problem. The APC approach is not suitable for the interaction parameter matrices in the current application since they are not sparse in the statistical sense as is the case with residue interaction matrices.

The pseudolikelihood method allows an efficient correction for population structure by the reweighting scheme used in plmDCA for an MSA in protein analysis[58], which ensures that highly similar sequences are not artificially inflating the support for direct dependence between alleles. We used the default reweighting scheme in plmDCA with a 0.9 similarity threshold over the variable alignment positions, except for the 5078 locus M1 data where the minimum similarity between any pair of sequences was 98% and threshold 0.95 was used.

### Resampling procedure

The number of parameters in our model for the pneumococcus data is far larger than has hereto been considered in analogous studies. Also, neighboring loci are most often in linkage disequilibrium (LD) which has a confounding biological effect on their interaction. For both these reasons, we have chosen to dissect the pneumococcal core chromosome into approximately 1,500 non-overlapping segments and apply pseudolikelihood inference on subsets of the loci, chosen randomly from each genomic window of average size of 1500 nt. These particular choices were motivated by attempting to achieve a balance between minimizing the effect of LD and the need to keep the number of SNP loci included in any single instance of the pseudolikelihood inference limited enough such that the computation time would remain reasonable. For each such sample we learned about 10^6^ parameters, scored them as described above and saved only the 3000 largest interaction parameters to reduce memory consumption. As seen from the results, the vast majority of the stored maximal couplings were relatively small, indicating that sufficiently many values were included from each analysis. For each sample the inference took approximately 190 minutes and used approximately 2GB of memory on a single core of a standard Intel Core i5 processor. The whole re-sampling and model fitting procedure was repeated 38,000 times to ensure stable inference about the parameters. However, sequential examination of the results from the estimation runs showed that the list of top scoring couplings did not change markedly after a few thousand iterations, indicating that substantially fewer iterations had been sufficient for practical purposes. Interaction estimates were averaged for any pairs of sites that occurred multiple times among the saved parameters, which resulted in 102,551 pairs of sites with non-negligible coupling coefficients from the aggregated re-sampling results (S1 Table). This set is approximately five orders of magnitude smaller than the set of all possible interactions for the 81,560 considered loci.

The whole pneumococcus analysis workflow is illustrated in S4 Fig. Software implementing both the resampling procedure (Python) and the parameter inference (Matlab) is made freely available at https://github.com/mskwark/genomeDCA. The smaller number of variable sites in the *S*. *pyogenes* alignment made it possible to fit the model simultaneously to all considered SNP loci and save the couplings exhaustively without resampling. The analysis of the smaller set of 324 loci took approximately 2 minutes and used 370 MB of memory, whereas the set of 5078 loci took approximately 11 hours and used 5GB of memory. The couplings for the smaller set are listed in S4 Table (52326 entries) and for the larger set in S6 Table (approximately 13M entries), which is available from the Dryad Digital Repository: http://dx.doi.org/10.5061/dryad.gd14g.

### Choice of significance threshold for interactions

To select a list of highest scoring interactions among the 102,551 estimates for the pneumococcus which are unlikely a result of neutral and sampling variation in the studied population, we employed the statistical theory of extreme value distributions [73]. Since in each resampling step the largest 3000 parameters were saved, these can under a null model of random interactions between loci be considered as a sample from an extreme value distribution, such as the Gumbel distribution. We fitted a Gumbel distribution to the distribution of the estimated parameters using least squares minimization between the fitted distribution and the empirical rank distribution of the coefficients located between 25% and 75% quantiles. Fig 1A shows the fitted distribution which has a remarkably good fit to the vast majority (95%) of the coefficients. To select a threshold for a significant deviation from the null model we identified the first value for which the predicted curve was more than six standard deviations (SD) away from the empirical distributions (Fig 1B). The SD was estimated using the deviances for the 50,000 smallest coefficients. The 5199 couplings exceeding the threshold 0.129 are listed in S2 Table and in addition the 500 strongest couplings in S3 Table. The latter were used in the structural plots for the PBPs. The same Gumbel model fitting procedure was also employed to define the significance thresholds for the *S*. *pyogenes* data, except that we excluded all couplings below 0.01 for the larger locus set where the vast majority of the nearly 13M couplings in total were very close to zero (S5 Table). This resulted in 7514 couplings being included in the Gumbel analysis. The empirical distributions and the fitted Gumbel models are shown in Fig 6.

A resampling-based analysis of haplotypes generated randomly from a population by merging alleles sampled from the marginal allele frequency distribution of each SNP locus showed that couplings as large as those exceeding the threshold chosen for the coefficients in the original data were never encountered (S3 Fig). In the analysis we used 5000 replicates of the haplotype re-sampling based on the same chromosomal windows as in the analysis of the original data. Hence, our approach was concluded to maintain a strict control of false positive interactions for unlinked loci stemming from population sampling variation.

### Functional analysis and structural modeling

Networks were displayed and analysed using Cytoscape [74]. GO terms were inferred from applying Interpro scan [75] and CD-search [44] to the *S*. *pneumoniae* ATCC 700669 genome [EMBL accession: FM211187]. These were matched to network components, and a Fisher exact test used to test for enrichment of 139 instances of GO terms that featured in a network component twice or more, relative to the CDSs that contained sites analyzed in this study, but not found to include a significantly coupled loci. The *p* values were corrected for multiple testing using the method of Benjamini and Hochberg [76].

Crystal structures of *S*. *pneumoniae* PBPs with the following IDs: 2C5W (*pbp1a*), 2WAF (*pbp2b*), 2ZC3 (*pbp2x*) were retrieved from the Protein Data Bank[77] (www.rcsb.org; accession date January 8, 2016) and visualized in The PyMOL Molecular Graphics System, Version 1.8 Schrödinger, LLC. Inferred co-evolving sites were visualized using the Circos software[78].

## Supporting information

S1 Text Mathematical details of the Potts model for DCA. (DOCX)

S1 Fig Comparing the distributions of coupled sites in penicillin-binding protein genes with those identified by a genome-wide association study for polymorphisms associated with beta lactam resistance. (A) Domain annotation of the PBP2X, PBP1A and PBP2B proteins, based on analysis with the Pfam database. (B) Distribution of coupled loci identified by this analysis. The columns corresponding to polymorphic loci identified as being significantly coupled with others by this analysis are coloured according to the base present in each isolate in the collection, ordered according to the whole genome phylogeny shown on the left. (C) Distribution of loci found to be significantly associated with beta lactam resistance through a genome-wide association study of this Maela population by Chewapreecha *et al*. Only those sites that match the inclusion criteria for this study (i.e. biallelic with <15% of sites missing across the population), and are within the three displayed genes, are shown. The phylogeny is estimated from all core genome SNPs for the 3,156 isolates using the GTR model with approximate rate heterogeneity in FastTree as in Chewapreecha *et al*.
(TIF)

S2 Fig Bipartite network showing the couplings between coding sequences and upstream untranslated regions. The network is displayed as in Fig 3, except that untranslated regions are shown as black triangles. (A) Network component showing the links between *pbp2x*, *pbp2b* and the upstream regions around *pbp1a*. (B) Network component showing the coupling between *smc* and upstream regions. (C) Network component showing the *pspA* and *divIVA* genes.
(TIF)

S3 Fig Distributions of estimated coupling coefficients for the original data and for haplotypes generated randomly from a population by merging alleles sampled from the marginal allele frequency distribution of each SNP locus. The red curve is generated from 5000 replicates of the haplotype re-sampling based on the same chromosomal windows as used in the analysis of the original data.
(TIF)

S4 Fig Workflow chart describing the inference procedure employed for the pneumococcus data. (TIF)

S5 Fig Empirical distribution functions of coupling values for loci including a SNP with either rare or more common MAF in the M1 data set. (TIF)

S6 Fig Comparison of rankings of the estimated couplings for the M1 324 locus set under either no population structure correction or a varying threshold for average sequence identity. (TIF)

S1 Table List of 102,551 estimated SNP couplings for the Maela data. (XLSX)

S2 Table List of 5,199 significant couplings for the Maela data. (XLSX)

S3 Table Genomic positions and estimated couplings for the inter-PBP links shown in Fig 5. (XLSX)

S4 Table Estimated couplings for 324 SNP loci with MAF > 1% in the *S*. *pyogenes* data. (TXT)

S5 Table Access numbers for the Maela data. (TXT)
