# Supplementary material for: Interacting networks of resistance, virulence and core machinery genes identified by genome-wide epistasis analysis
Source: PLoS Genet. 2017 Feb 16;13(2):e1006508. doi: 10.1371/journal.pgen.1006508 (PMC5312804; doi:10.1371/journal.pgen.1006508)
Supplement: S1 Text — (DOCX) [file pgen.1006508.s001.docx]

**Mathematical definition and notational details on Potts models**:

Let $\left( s_{1},s_{2}\ldots,s_{N} \right)$ be a string of N symbols, each of which can take values 0, 1 or 2. A *q=3* Potts model is a probability distribution on such strings defined by the following formula

$$P\left( s_{1},s_{2},\ldots,s_{N} \right)=\frac{1}{Z}e^{E\left( s_{1},s_{2},\ldots,s_{N} \right)}$$

where *Z* is a normalization constant called the *partition function*, and the function in the exponent is given by

$$E\left( s_{1},s_{2},\ldots,s_{N} \right)= \sum_{i=1}^{N} \sum_{a=0}^{2} h_{i}\left( a \right)\delta_{s_{i},a}+\sum_{i,j=1}^{N} \sum_{a,b=0}^{2} J_{ij}\left( a,b \right)\delta_{s_{i},a}\delta_{s_{j},b}$$

In above $\delta_{x.y}$ is the Kronecker symbol which is equal to one if *x* and *y* are equal and otherwise equal to zero. The *linear terms* referred to in the main text are $h_{i}\left( a \right)\delta_{s_{i},a}$ for different values of *i* and *a*. The coefficients $h_{i}\left( a \right)$ (3N real numbers) are sometimes referred to as *external fields*; they parametrize a bias for each variable to take a given value, independently of the values of all the others variables. The quadratic terms referred to in the main text are $J_{ij}\left( a,b \right)\delta_{s_{i},a}\delta_{s_{j},b}$ for different values of *i* and *j,* and *a* and *b*. The coefficients $J_{ij}\left( a,b \right)$, which are the *couplings* or *interactions* of Direct Coupling Analysis (DCA), are taken to be zero when the two indices *i* and *j* are equal (the same effect is then parametrized by the external fields) and are symmetric in the interchanges $i\leftrightarrow j$ and $a\leftrightarrow b$. The *q=3* Potts model thus contains $\frac{9N(N-1)}{2}$ real parameters specifying the *J*’s.

It is easy to see that the above constitutes an over-parametrization of the model in the sense that two different sets of parameters *h* and *J* can yield the same probability distribution *P*. However, it is also easy to see that this over-parametrizations can be eliminated by restricting certain sums of parameters to be zero. This issue has been discussed in detail in the DCA literature, *e.g*. by one of us in^2^. Potts models after elimination of over-parametrization can hence be written as above with some constraints on the allowed values of the parameters.

For the special case of the Ising model (Potts model with *q=2*) one can also write the probability distribution in a simpler way as

$$P\left( s_{1},s_{2},\ldots,s_{N} \right)=\frac{1}{Z}e^{\sum_{i=1}^{N} h_{i}s_{i}+ \sum_{i,j=1}^{N} J_{ij}s_{i}s_{j}}$$

where each $h_{i}$ and each $J_{ij}$ is just one real number. The elimination of the over-parametrization thus brings down the number of external field parameters in an Ising model from *2N* to *N* and the number of coupling parameters from $\frac{4N(N-1)}{2}$ to $\frac{N(N-1)}{2}$.

The log-likelihood function of *n* independent samples of the Potts model (q=3) is

$$L\left[ \underline{s}^{1},\ldots,\underline{s}^{n} \right]=\frac{1}{n}\sum_{r=1}^{n} \left( \sum_{i=1}^{N} \sum_{a=0}^{2} h_{i}\left( a \right)\delta_{s_{i}^{r},a}+\sum_{i,j=1}^{N} \sum_{a,b=0}^{2} J_{ij}\left( a,b \right)\delta_{s_{i}^{r},a}\delta_{s_{j}^{r},a} \right)-log Z$$

where $s_{i}^{r}$now indicates the value of variable *i* in sample *r* and $\underline{s}^{r}$ all the variables in sample *r*. As discussed in the main text it is not computationally feasible to maximize this log-likelihood, and in the pseudo-likelihood inference method one instead maximizes the following set of auxiliary functions

$${PL}_{i}\left[ \underline{s}^{1},\ldots,\underline{s}^{n} \right]=\frac{1}{n}\sum_{r=1}^{n} \left( h_{i}\left( s_{i}^{r} \right)+\sum_{j=1}^{N} J_{ij}\left( s_{i}^{r},s_{j}^{r} \right)-f_{i}\left[ \underline{s}^{r} \right] \right)$$

The interpretation of ${PL}_{i}\left( a \right)$ is that it is $\frac{1}{n}$ times the logarithm of the product of the conditional probabilities of variable *i* to take the observed values $s_{i}^{r}$in each sample *r*, conditioned by the values $s_{\backslash i}^{r}$of all the other observed variables in the same sample. The numbers $f_{i}$ are hence normalizations of these conditional probabilities, and explicitly given by

$$f_{i}\left[ \underline{s}^{r} \right]=log\left( \sum_{a=0}^{2} e^{h_{i}\left( a \right)+\sum_{j=1}^{N} J_{ij}\left( a,s_{j}^{r} \right)} \right)$$

The arguments of the maximization of ${PL}_{i}$ are the *q* external fields $h_{i}\left( a \right)$ and the $q^{2}\left( N-1 \right)$couplings $J_{ij}\left( a,b \right)$. Optimization is not well-defined for sufficiently under-sampled problems, and regularization is necessary. We have here used $L_{2}$ regularization as in the earlier developed method plmDCA (asymmetric version)^1-3^, using values of the regularization parameters as set by the program. As discussed in Ekeberg et al.^2^ combining maximization of ${PL}_{i}\left( a \right)$ and $L_{2}$ regularization lifts the over-parametrization discussed above.

Finally, in re-weighting, as described in the main text, each sample *r* is assigned a weight $w_{r}$such that $\sum w_{r}=1.$ The function to be optimized is then modified to

$${PL}_{i}\left[ \underline{s}^{1},\ldots,\underline{s}^{n} \right]=\sum_{r=1}^{n} w_{r}\left( h_{i}\left( s_{i}^{r} \right)+\sum_{j=1}^{N} J_{ij}\left( s_{i}^{r},s_{j}^{r} \right)-f_{i}\left[ \underline{s}^{r} \right] \right)$$

References:

^1^M. Ekeberg, C. Lövkvist, Y. Lan, M. Weigt, E. Aurell, Improved contact prediction in proteins: Using pseudolikelihoods to infer Potts models, Phys. Rev. E 87, 012707 (2013).

^2^M. Ekeberg, T. Hartonen, E. Aurell, Fast pseudolikelihood maximization for direct-coupling analysis of protein structure from many homologous amino-acid sequences, J. Comput. Phys. 276, 341-356 (2014).

^3^https://libraries.io/github/magnusekeberg/plmDCA
