# Supplementary figures and images for: Interacting networks of resistance, virulence and core machinery genes identified by genome-wide epistasis analysis
Source: PLoS Genet. 2017 Feb 16;13(2):e1006508. doi: 10.1371/journal.pgen.1006508 (PMC5312804; doi:10.1371/journal.pgen.1006508)

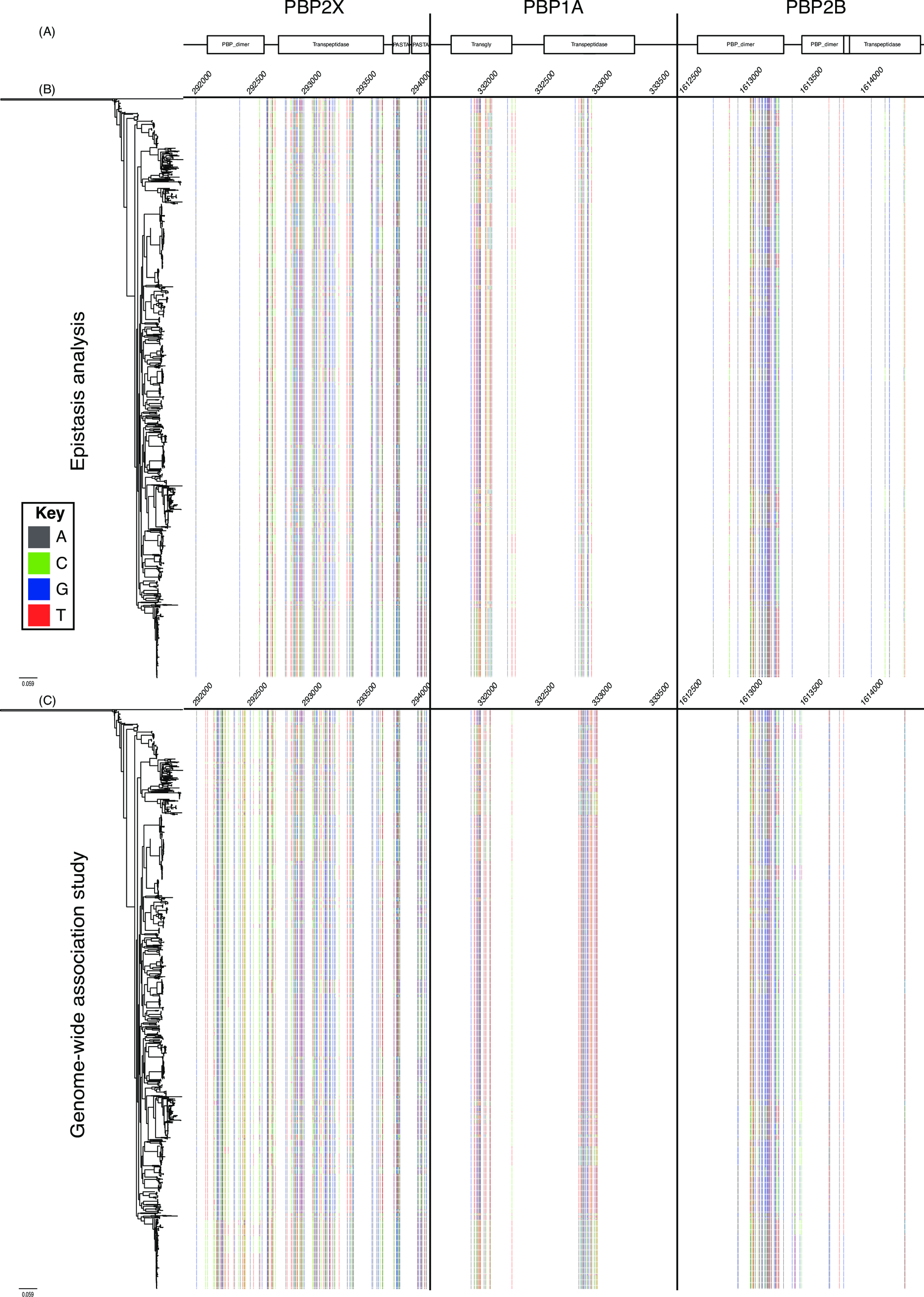

Supplement: S1 Fig — (A) Domain annotation of the PBP2X, PBP1A and PBP2B proteins, based on analysis with the Pfam database. (B) Distribution of coupled loci identified by this analysis. The columns corresponding to polymorphic loci identified as being significantly coupled with others by this analysis are coloured according to the base present in each isolate in the collection, ordered according to the whole genome phylogeny shown on the left. (C) Distribution of loci found to be significantly associated with beta lactam resistance through a genome-wide association study of this Maela population by Chewapreecha et al. Only those sites that match the inclusion criteria for this study (i.e. biallelic with <15% of sites missing across the population), and are within the three displayed genes, are shown. The phylogeny is estimated from all core genome SNPs for the 3,156 isolates using the GTR model with approximate rate heterogeneity in FastTree as in Chewapreecha et al. (TIF) [file pgen.1006508.s002.tif]

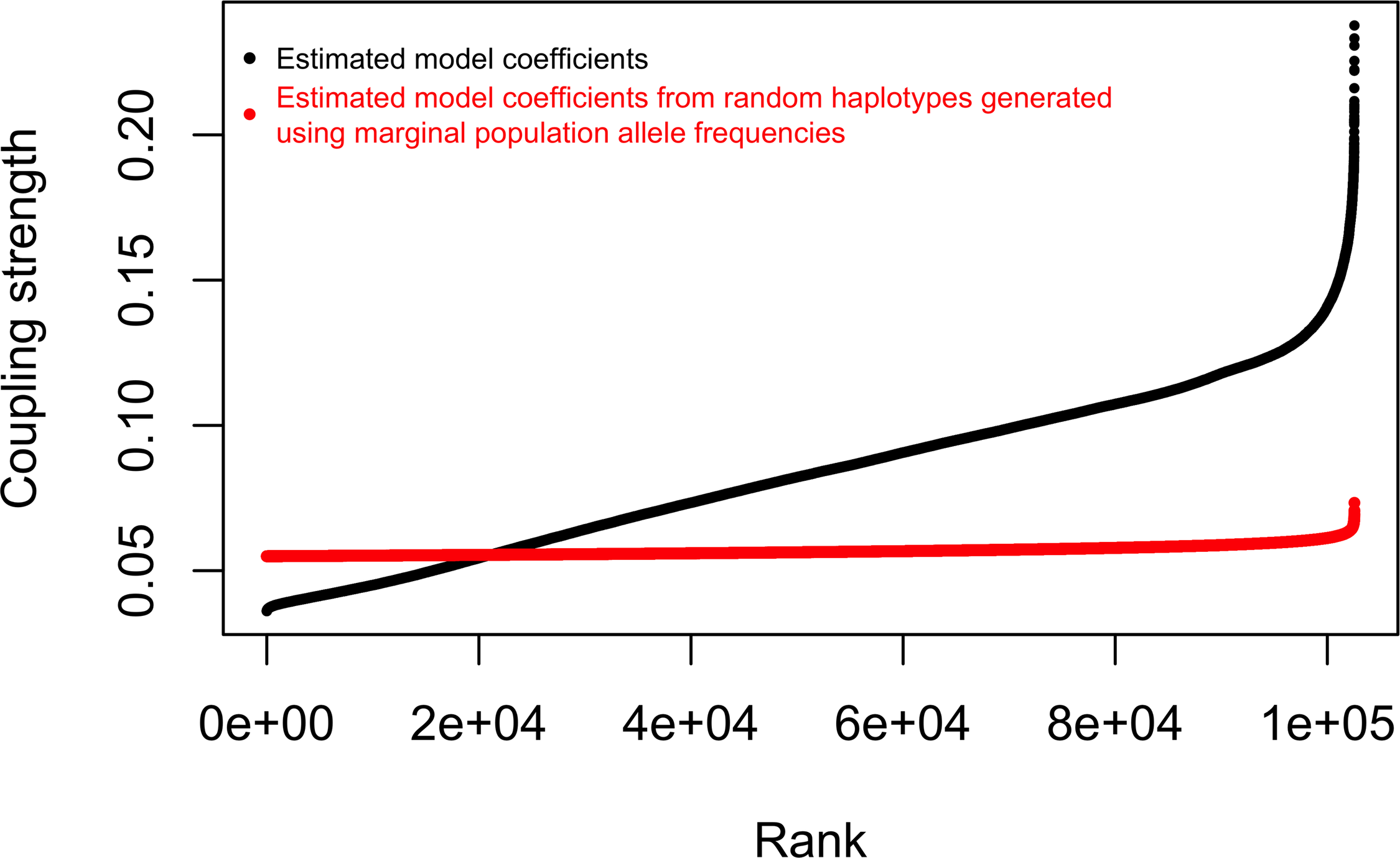

Supplement: S3 Fig — The red curve is generated from 5000 replicates of the haplotype re-sampling based on the same chromosomal windows as used in the analysis of the original data. (TIF) [file pgen.1006508.s004.tif]

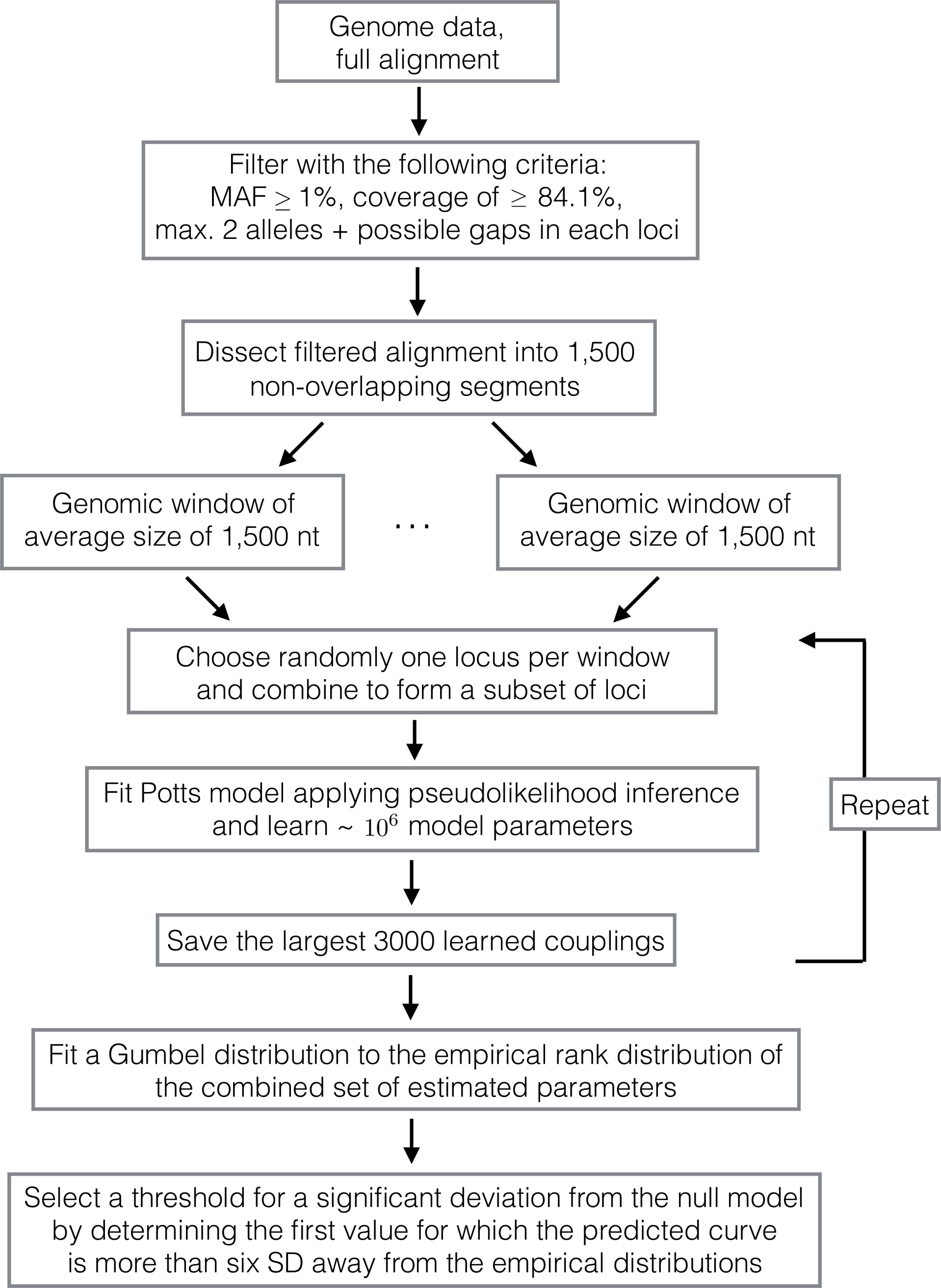

Supplement: S4 Fig — (TIF) [file pgen.1006508.s005.tif]

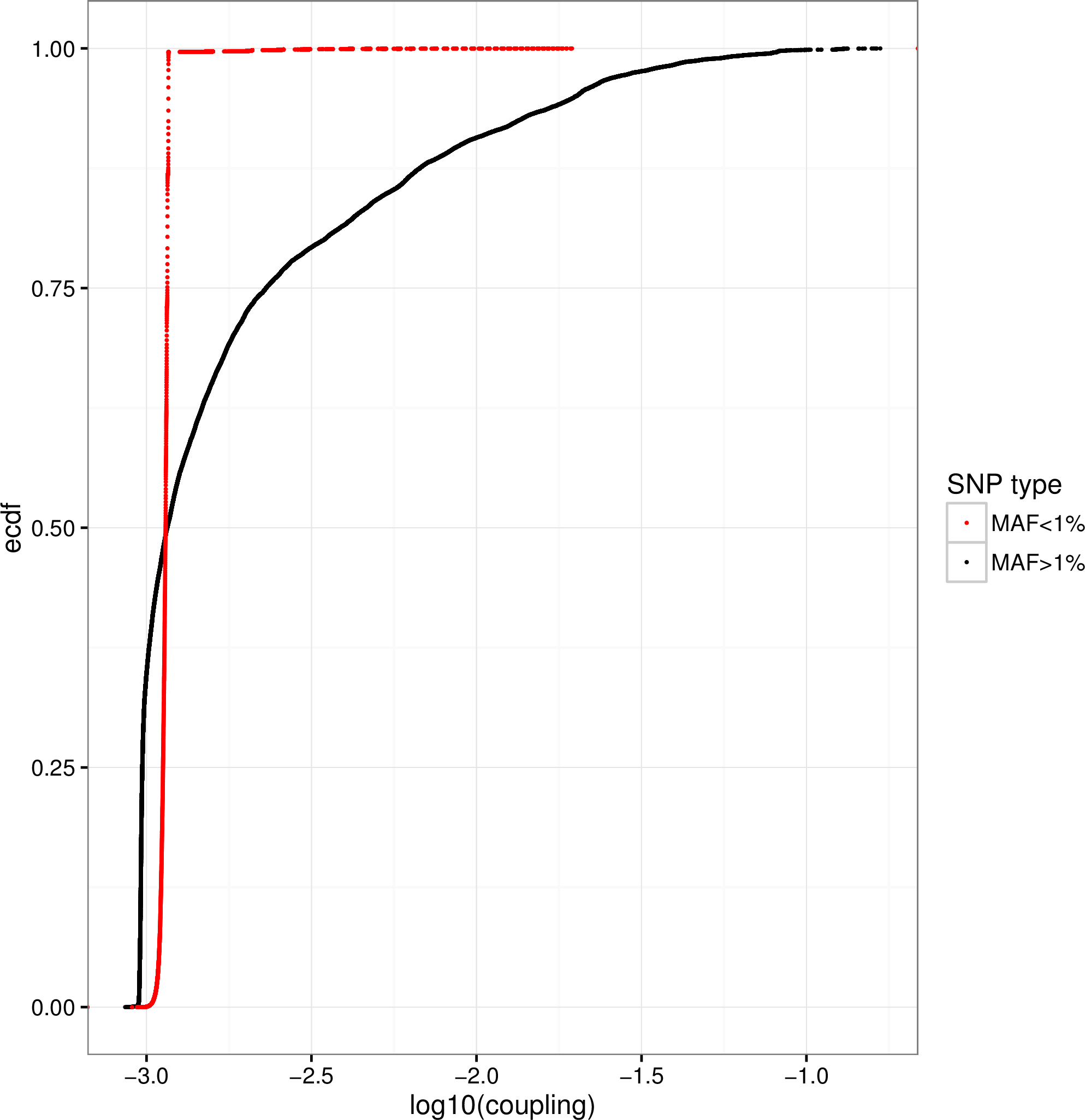

Supplement: S5 Fig — (TIF) [file pgen.1006508.s006.tif]

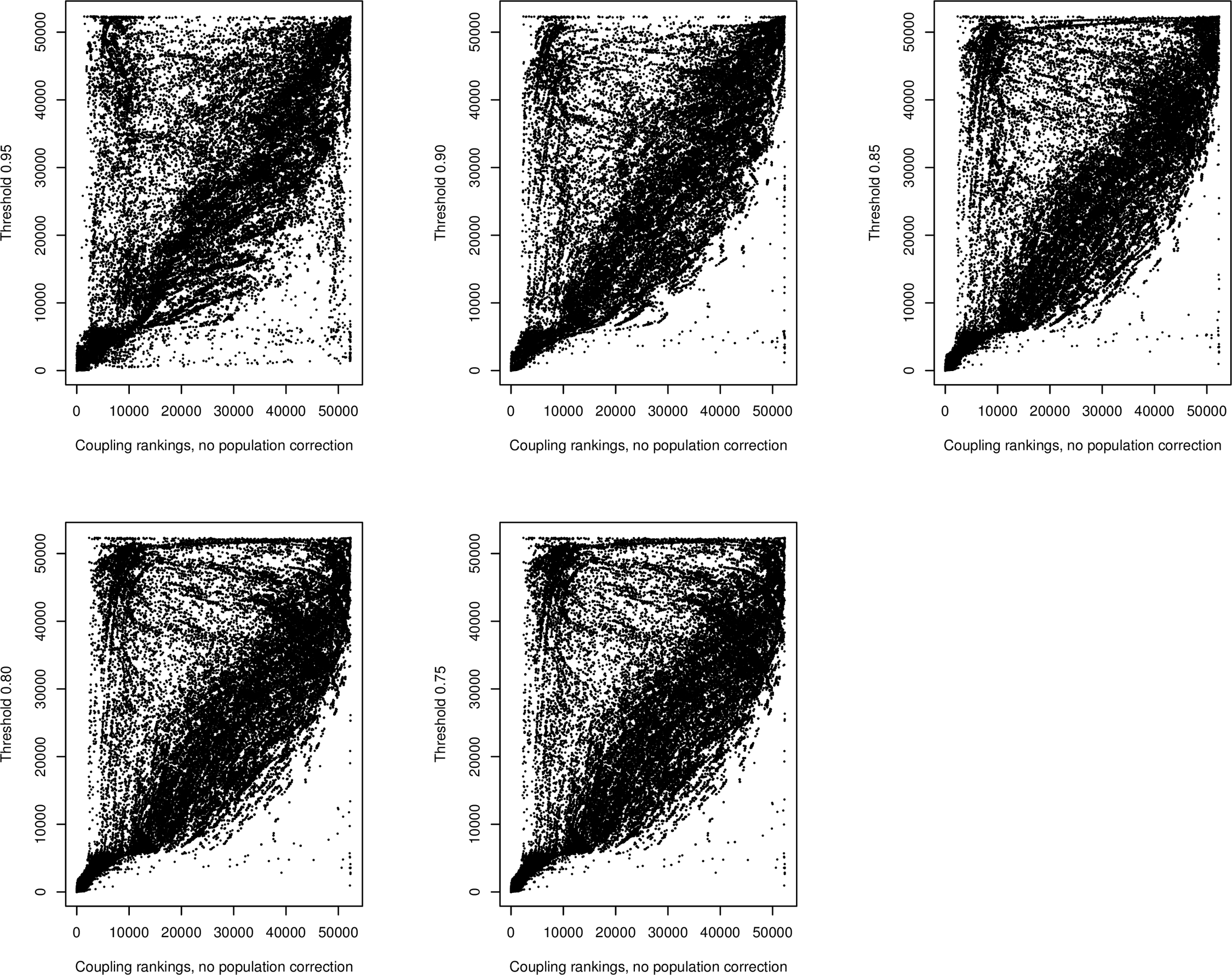

Supplement: S6 Fig — (TIF) [file pgen.1006508.s007.tif]
